# Large-scale identification of polymorphic microsatellites using an *in silico *approach

**DOI:** 10.1186/1471-2105-9-374

**Published:** 2008-09-15

**Authors:** Jifeng Tang, Samantha J Baldwin, Jeanne ME Jacobs, C Gerard van der Linden, Roeland E Voorrips, Jack AM Leunissen, Herman van Eck, Ben Vosman

**Affiliations:** 1 Wageningen UR Plant Breeding, PO Box 16, 6700 AA Wageningen, the Netherlands; 2 Laboratory of Bioinformatics, Wageningen University, PO Box 8128, 6700 ET Wageningen, the Netherlands; 3 New Zealand Institute for Crop and Food Research, Private Bag 4704, Christchurch, New Zealand

## Abstract

**Background:**

Simple Sequence Repeat (SSR) or microsatellite markers are valuable for genetic research. Experimental methods to develop SSR markers are laborious, time consuming and expensive. *In silico *approaches have become a practicable and relatively inexpensive alternative during the last decade, although testing putative SSR markers still is time consuming and expensive. In many species only a relatively small percentage of SSR markers turn out to be polymorphic. This is particularly true for markers derived from expressed sequence tags (ESTs). In EST databases a large redundancy of sequences is present, which may contain information on length-polymorphisms in the SSR they contain, and whether they have been derived from heterozygotes or from different genotypes. Up to now, although a number of programs have been developed to identify SSRs in EST sequences, no software can detect putatively polymorphic SSRs.

**Results:**

We have developed PolySSR, a new pipeline to identify polymorphic SSRs rather than just SSRs. Sequence information is obtained from public EST databases derived from heterozygous individuals and/or at least two different genotypes. The pipeline includes PCR-primer design for the putatively polymorphic SSR markers, taking into account Single Nucleotide Polymorphisms (SNPs) in the flanking regions, thereby improving the success rate of the potential markers. A large number of polymorphic SSRs were identified using publicly available EST sequences of potato, tomato, rice, *Arabidopsis*, *Brassica *and chicken.

The SSRs obtained were divided into long and short based on the number of times the motif was repeated. Surprisingly, the frequency of polymorphic SSRs was much higher in the short SSRs.

**Conclusion:**

PolySSR is a very effective tool to identify polymorphic SSRs. Using PolySSR, several hundred putative markers were developed and stored in a searchable database. Validation experiments showed that almost all markers that were indicated as putatively polymorphic by polySSR were indeed polymorphic. This greatly improves the efficiency of marker development, especially in species where there are low levels of polymorphism, like tomato. When combined with the new sequencing technologies PolySSR will have a big impact on the development of polymorphic SSRs in any species.

PolySSR and the polymorphic SSR marker database are available from .

## Background

Microsatellites, or simple sequence repeats (SSRs), are tandem repeats of 1–6 nucleotides and are present in all eukaryotic genomes. Based on their locus-specificity, high level of polymorphism (due to their multi-allelic nature), co-dominant inheritance, relative abundance and reproducibility, SSRs have become valuable tools for genetic mapping, association mapping, comparative mapping, diversity analysis, and QTL analysis [1-3].

The large number of expressed sequence tags (ESTs) deposited in public databases is a valuable resource to develop SSR markers. Moreover, EST-derived SSRs (EST-SSRs) can be more readily transferred between (related) species relative to SSRs obtained from random genomic sequences [3-5]. Some previous studies indicated that EST-SSRs are less polymorphic than genomic SSRs [6-8], while others suggest the rate of polymorphism in EST-SSRs was similar to that of genomic SSRs [9,10].

Conventional experimental methods [11] for developing SSRs are laborious, time consuming and expensive. Meanwhile, with the ever increasing number of sequences in public databases, *in silico *approaches to screen for SSRs have become a practicable and inexpensive alternative for many species. Several software packages have been developed to detect SSRs in DNA sequences, especially in ESTs [3,12,13]). Public EST databases may contain redundancy in sequences of a particular gene, e.g. different alleles derived from heterozygous individuals or from different genotypes. Some redundant sequences can contain information on length-polymorphisms in SSRs. In the past this information was often lost due to the elimination of sequence redundancy before SSR identification [10,14-16]. Only very recently, by comparing the genomic sequences of the two rice subspecies *indica *and *japonica*, have polymorphic SSRs been detected successfully [17]. Also, Feingold et al. [18] recognized the length-polymorphisms present in the EST database and manually identified polymorphic SSRs. Up to now there are no software packages that can identify polymorphic SSRs from ESTs in an automated way. Our research aims to develop a new tool, called PolySSR, for the identification of polymorphic SSRs using EST sequence redundancy. The versatility of the tool was shown in a number of species (potato, tomato, *Brassica*, rice, *Arabidopsis *and chicken).

## Results

### Identification of polymorphic SSR

A substantial frequency of SSRs in EST sequences were predicted by Sputnik [13] using the default settings, and ranged from 9% (*Brassica*) to 28% (rice) (Table 1). Clustering of EST sequences using CAP3 with 95% similarity over 100 nucleotides resulted in clusters and a large number of singletons, both of which may contain SSRs (Table 1). In the non-redundant sequences of the species, between 13% (tomato) and 27% (rice) contained SSRs, and between 18% (tomato) and 43% (rice) of clusters contained an SSR. When using PolySSR between 7% (tomato) and 22% (potato) of the SSRs in the clusters were found to be polymorphic. For almost all of the predicted polymorphic SSRs, primers could be designed that allow PCR amplification of the SSR (Table 1; our website [19]) resulting in 263 putative polymorphic markers for tomato, 1,053 for potato, 937 for *Brassica*, 1,163 for *Arabidopsis*, 2,555 for rice and 1,667 for chicken. These polymorphic SSRs can either be perfect (no point mutations within the SSR) or imperfect (with one or a few point mutations in the SSR) (See Materials and Methods).

**Table 1:** Number of ESTs, clusters, SSRs and polymorphic SSRs of chicken, rice, *Arabidopsis*, *Brassica*, potato and tomato

	chicken	rice	*Arabidopsis*	*Brassica*	potato	tomato
ESTs	599,330	1,211,078	734,275	163,750	219,765	249,794
Non-redundant sequences^1^	283,434	493,818	224,994	58,260	72,381	54,182
Clusters^1^	44,654	35,154	33,052	20,468	25,228	21,229
Singletons^1^	238,780	458,664	191,942	37,792	47,153	32,953
ESTs with SSRs (%)^2^	74,297 (12%)	336,569 (28%)	127,757 (17%)	14,968 (9%)	29,481 (13%)	28,728 (11%)
SSRs Non-redundant sequences (%)^2^	40,020 (14%)	133,861 (27%)	38,096 (17%)	13,251 (23%)	10,537 (15%)	7,163 (13%)
Singletons with SSRs (%)^2^	31,119 (13%)	118,649 (26%)	29,843 (16%)	7,328 (19%)	5,717 (12%)	3,261 (10%)
SSR in clusters (%)^2^	8,901 (20%)	15,212 (43%)	8,253 (25%)	5,923 (29%)	4,820 (19%)	3,902 (18%)
Polymorphic SSRs^3 ^(%)	1,724 (19%)	2,646 (17%)	1,248 (15%)	997 (17%)	1,080 (22%)	265 (7%)
Polymorphic SSRs with primers (%)	1,667 (97%)	2,555 (97%)	1,163 (93%)	937 (94%)	1,053 (97%)	263 (99%)
% polymorphism in long SSRs^4^	15%	11%	12%	13%	17%	6%
% polymorphism in short SSRs^5^	36%	23%	30%	40%	46%	14%

^1 ^clusters and singletons were produced using CAP3 with 95% similarity for 100 nucleotides overlaps; 'non-redundant sequences' includes clusters and singletons

^2 ^SSR detected by Sputnik [13] using default settings

^3^polymorphic SSRs detected by PolySSR (settings used are described in Materials and Methods); % = percentage of SSRs in clusters that are polymorphic SSRs.

^4 ^long polymorphic SSRs are SSRs with at least 10 repeats for dinucleotide SSRs, 6 repeats for trinuleotide SSRs, 5 repeats for tetra-, penta- or hexanucleotide SSRs; % = percentage of polymorphic SSRs in long SSRs;

^5^short polymorphic SSRs are SSRs with a maximum of 5 repeats for dinucleotide SSRs, and 4 repeats for tri-, tetra-, penta- and hexanucleotide SSRs; the minimum number of repeats was in this case set to 3 for all SSR types; % = percentage of polymorphic SSR in short SSRs

### Experimental validation of potentially polymorphic SSRs

We first performed a validation experiment using tomato data from the scientific literature. Tomato is a self-pollinating crop with very little genetic variation between cultivars [20,21]. Forty-six of the 75 EST-derived SSRs from He et al. [21] were also present among the 3,902 SSRs identified by PolySSR in tomato (Table 1). Of these 46 SSRs, 16 were polymorphic and 30 monomorphic according to He et al. [21]. The same classification was obtained using PolySSR for all 16 polymorphic, and for 29 of the 30 monomorphic SSRs.

We also selected some potato SSRs identified by PolySSR for validation. Potato is a tetraploid, cross-pollinating crop, with considerable allelic variation both within and between cultivars [18,22]. We randomly selected a set of 25 short polymorphic SSRs and 25 long polymorphic SSRs from the 1,053 potato polymorphic SSRs, as identified by PolySSR. In addition, a group of 30 SSRs, predicted to be monomorphic in the available EST dataset, were selected from the 3,740 putatively monomorphic SSRs. These include 15 short and 15 long monomorphic SSR (Table 2). All 50 polymorphic and 30 monomorphic SSRs were also proposed by TIGR [23]. Primers for these SSRs were taken from the TIGR database and evaluated using five potato cultivars including Bintje, Shepody and Kennebec, that had contributed most of the EST present in the database. Almost all the potato SSRs identified as putatively polymorphic, were indeed polymorphic (Table 2). In addition, most SSRs that represented monomorphic SSRs using the current EST set turned out to be polymorphic. The results from short and long SSRs were similar.

**Table 2:** Results of experimental validation of predicted long and short, polymorphic and monomorphic EST-SSRs of potato

Number of SSRs	long poly^2a^	short poly^2b^	long mono^3a^	short mono^3b^	total
for which primers were designed	25	25	15	15	80
with no or not clear products	2	4	6	1	13
with products more than 500 bp	2	2	1	2	7
that produced scorable markers^1^	21 (84%)	19 (76%)	8 (53%)	12 (80%)	60 (75%)
N of polymorphic SSRs	21 (100%)	18 (95%)	7 (88%)	10 (83%)	56 (93%)

^1^Marker are considered scorable when they produced amplicons of less than 500 base pairs

^2 ^EST-SSRs classified as polymorphic by PolySSR; ^3 ^EST-SSRs classified as monomorphic by PolySSR; ^a^long SSRs: at least 10 repeats in repeat motif for dinucleotide SSRs, 6 for tri-SSRs, 5 for tetra-, penta- and hexa-SSRs; ^b^short SSRs: at most 5 repeats for dinucleotide SSRs, 4 for tri-, tetra-, penta- and hexa-SSRs (see Materials and Methods).

### Position of the polymorphic SSRs in the expressed sequences

Table 3 shows the type and position of the polymorphic SSRs in the EST sequences. From this table it can be seen that the vast majority of all simple sequence repeats are present in the untranslated regions (UTRs). All repeat types, except the trinucleotide repeats, are found at higher frequencies in these regions. Trinucleotide repeats are mainly found in the coding regions. However, a substantial number of di- and pentanucleotide repeats is also found in this region. In most species, SSRs in the 5' UTR are more frequent than in the 3' UTR. To make a comparison between the different regions of the EST we calculated the polymorphic SSR density by taking the number of polymorphic SSRs per 100 kb. From Table 3 it can be seen that the overall density of polymorphic SSRs seems to be consistent across all species studied. It varied from 71 polymorphic SSRs per 100 kb in chicken to 119 SSRs per 100 kb in *Brassica*. When the different regions of the gene sequence were analyzed the UTRs and coding regions differed in polymorphic SSR density. The coding regions have a polymorphic SSR density which is on average 33% lower (chicken 23% – *Brassica *47%) than the overall density. The 5' UTR has an elevated polymorphic SSR density of 2.4 times higher than the overall polymorphic SSR density. The 3' UTR has a polymorphic SSR density similar to the all gene sequence density of SSR polymorphisms. Interestingly a few polymorphic SSR were found around the first codon (translation start) or last codon (translation stop) in every species studied.

**Table 3:** Motif length and position of polymorphic SSRs in EST sequences of chicken, rice, *Arabidopsis*, *Brassica*, potato and tomato

species		Di^a^-	Tri-^a^	Penta^a^	Others^b^	total	Density^c^
chicken	all	846 (49%)	378 (22%)	386 (22%)	114 (7%)	1724 (100%)	71.51
	coding	95	70	38	9	212	55.13
	5'UTR^1^	19	33	24	10	86	137.66
	3'UTR^2^	155	54	77	24	310	74.81
	TSS^3^	1	3	3	1	8	

rice	all	1070 (40%)	1113 (42%)	320 (12%)	143 (5%)	2646 (100%)	93.47
	coding	154	372	86	26	638	57.33
	5'UTR^1^	294	285	69	32	680	227.16
	3'UTR^2^	342	121	70	36	569	92.66
	TSS^3^	5	0	1	1	7	

*Arabidopsis*	all	668 (53%)	410 (33%)	133(11%)	37 (3%)	1248 (100%)	102.63
	coding	196	222	34	13	465	72.84
	5'UTR^1^	193	73	34	7	307	215.96
	3'UTR^2^	136	42	40	10	228	112.02
	TSS^3^	6	2	1	1	10	

*Brassica*	all	468 (47%)	350 (35%)	139 (14%)	40 (4%)	997 (100%)	119.45
	coding	55	203	43	13	314	63.09
	5'UTR^1^	205	55	27	13	300	378.62
	3'UTR^2^	126	44	47	9	226	169.90
	TSS^3^	1	2	3	2	8	

potato	all	379 (35%)	358 (33%)	248 (23%)	95 (9%)	1080 (100%)	89.78
	coding	61	155	87	44	347	62.66
	5'UTR^1^	64	38	29	8	139	160.21
	3'UTR^2^	129	40	59	24	252	134.75
	TSS^3^	0	1	3	1	5	

tomato	all	124 (47%)	79 (30%)	54 (20%)	8 (3%)	265 (100%)	98.52
	coding	42	35	19	2	98	67.06
	5'UTR^1^	50	18	19	1	88	265.74
	3'UTR^2^	12	7	5	0	24	70.79
	TSS^3^	1	1	1	1	4	

^a^Di- is dinucleotide SSR, and so on for others; ^b^others means tetranucleotide and hexanucleotides repeats or higher

^c^Density of number of SSR per 100 kb of EST sequences (See Materials & Methods).

^1 ^5' UTR is the portion of an mRNA from the 5' end to the position of the first codon used in translation.

^2^The 3' UTR is the portion of an mRNA from the last codon used in translation to the 3' end of the mRNA.

^3 ^TSS is the position of the first codon (translation start) and the last codon (translation stop) used in translation. SSR contain the first codon or the last codon.

### Effect of SSR length on frequency of polymorphism

We also studied the effect of the length of the SSRs on the frequency of polymorphism. For this the SSRs were divided in long and short (for details see Materials and Methods). The frequency of polymorphic SSRs depended on the species and on the length of the SSR, ranging from as low as 6% for long SSRs in tomato up to 46% for short SSRs in potato (Table 1). The frequency of polymorphic SSRs was higher for short SSRs than for long SSRs in all the species studied (Table 1).

## Discussion

### Features of PolySSR

PolySSR has two features that are important advances on earlier EST-SSR detecting software: 1) it is able to predict polymorphic SSRs, and 2) it is able to design high-quality primers for PCR amplification of polymorphic SSRs.

The first feature eliminates a lot of work by identifying and separating the many 'monomorphic' SSRs from the minority of polymorphic ones (7–22% in the species studied, Table 1). Although the frequency of identifying polymorphic EST-SSRs depends on the species and the genotypes used for the evaluation, previous research indicates that a large proportion of EST-SSR are not polymorphic [6,9]. As more EST sequences become available, the more pronounced the power of PolySSR will be to detect polymorphic SSRs.

**Figure 1:**
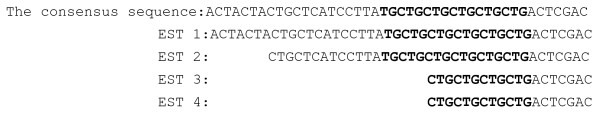
**An example of unreliable polymorphic SSRs**. Since the repeat chain in EST 3 and 4 does not extend to the end it is not clear whether these two ESTs represent a different (shorter) allele of the SSR or not. For that reason a minimum length for the flanking sequence used must be specified to reliably detect polymorphic SSRs.

The second feature takes into account a criterion that is important for the reliability of PCR amplification: the quality of flanking sequences [15]. This is of particular importance for EST-SSRs, since EST sequences are usually of poor quality, especially at the beginning and end of the sequence. Also, it is important that flanking sequences are of sufficient length to reduce possible artifacts (like the EST 3 and 4 in Figure 1; see also Materials and Methods). PolySSR uses at least 25 nucleotides on both sides of the SSR to filter out SSRs with low quality flanking sequences. Furthermore, potential single nucleotide polymorphisms (SNPs) identified by PolySSR are taken into account when designing primers. This is accomplished by changing the SNPs in the consensus sequence of a contig into N's. Primer3 [24] excludes these positions as suitable positions for primers. Primer sequences of potato SSRs, as provided by TIGR do not take into account potential SNPs around the SSR. Some of these primers are in regions where SNPs are present and therefore may produce unreliable amplicons in some genotypes. PCR primers that fail to anneal to the DNA template will result in null-alleles, which are difficult to deal with in genetic experiments. It is also possible that a SSR predicted to be polymorphic becomes monomorphic because the primers amplify one allele only. Using the improved strategy we were able to design reliable primers for more than 93% of the polymorphic SSR in *Arabidopsis*, *Brassica*, rice, potato, chicken and tomato (Table 1).

### Consequences of using CAP3 for clustering

The sequence assembly program CAP3 used in this study generated more gene clusters and singletons than expected. For the model species rice, using 95% similarity for 100 nucleotides overlap of CAP3, 35,154 gene clusters were generated and 458,664 ESTs were excluded from any cluster. Even using the default setting (75% similarity for 30 nucleotides overlap), 32,001 clusters and 303,900 singletons are produced. However, the total number of expressed genes of rice is estimated to be 30,000 – 60,000 [25,26]. The Rice Gene Index of TIGR contains 77,158 clusters and 85,212 singletons that were created from 1,278,120 sequences on June 20^th ^2006. The sequence assembling protocols used in TIGR and in our study try to assemble sequences under the criterion that sequences should match end – to-end with a certain "identity". However, it is not uncommon that both ends of an EST sequence are of poor quality. As a consequence of insufficient overlaps and/or poor quality overlaps, some sequences (alleles) from the same gene may not be assembled into the same cluster. This will result in overestimation of the number of clusters and singletons. Similar situations were found during analysis of *Arabidopsis *and apple ESTs [27]. As a consequence, this may result in an underestimation of the level of polymorphism in SSRs. In our study we found evidence for this. For example, in potato the polymorphic SSR st4913 was also found in another polymorphic cluster (3805). The same was found for the polymorphic SSR st13093 and st14911, which were also present in the clusters 15317 and 18454, respectively. In addition a non-polymorphic SSR (st2988) was found polymorphic in cluster 22236 (see additional file 1). At present it is not clear whether this is caused by the redundancy between clusters or by the presence of paralogous genes. Also Feingold et al [18] observed size variants of several SSRs that were not placed in the same clusters in the TIGR potato SSR database. If this occurs it may cause repetitive validation of the same SSR or PCR amplification of paralogs. To prevent this, we developed a program called CheckSSR (see Materials and Methods) to examine the presence of redundancy and paralogs in our SSR database. The program checks whether a particular SSR is unique. To accomplish this it compares primer sequence to the consensus sequence of each cluster and to all singleton sequences.

### Performance of PolySSR

Sixteen of the 32 polymorphic tomato SSRs identified by He et al. [21] were also present in our database of polymorphic tomato EST SSRs. The fact that this is only 50% may be caused by the limited number of EST sequences available in the EST database and by the limited number of genotypes contributing to the ESTs. Of the monomorphic SSRs identified by He et al. [21] only one of the 30 was found to be polymorphic in our data set; all others were indeed monomorphic. This single polymorphic microsatellite was derived from a genotype that was not used by He et al. [21]. The sequences containing the second allele of the SSR (DB684931 and DB696229, [28]) were from genotype Micro-Tom, a tomato breed for experimental research rather than a commercial cultivar.

One of the 40 tested potato SSRs predicted to be polymorphic (st13093) showed no polymorphism in our set of cultivars. Based on the EST, it was expected to be polymorphic both within and between varieties Kennebec, Bintje and Shepody (see website cluster ID 13093), with a difference of 3 base pairs between the two alleles. A monomorphic fragment was obtained from Kennebec and Bintje, and no amplification products at all for Shepody. Careful examination of the cluster indicated that the observed 3 base pair difference in SSR length was compensated for by a 3 base pair insertion associated with the shortest variant of the SSR between one of the primers and the SSR, which resulted in fragments of equal length for all cultivars. An explanation for the non-amplification in Shepody may be related to the relatively high variability of DNA regions directly flanking the SSR. In such regions SNPs and short indels occur frequently [29]. The reverse primer that we used was located close to the SSR and perhaps targetting an indel.

Of the 80 tested potato SSRs, 13 generated either no or no clear amplification products and 7 others produced amplicons larger than 500 base pairs (Table 2). Amplicons that are larger than predicted are likely to be the result of the presence of introns, while the presence of large introns can result in no amplification at all. In tetraploid and heterozygous potato cultivars, a maximum of 4 alleles per marker is expected [22]. When more alleles are found, this is most likely due to the amplification of paralogous sequences in addition to the target sequence. Evidence for the latter was found for 4 tested markers that produced more than the expected maximum of 4 alleles in some genotypes (see additional file 1).

From the 40 SSRs that produced clear patterns, 39 SSRs indeed showed the expected polymorphisms (Table 2). Compared to the highest reported success rate of EST-SSRs in potato (65%) [18] this is still a considerable improvement. The SSRs predicted to be monomorphic also resulted in the detection of a large number of polymorphic SSRs (17 out of 20, 85%). This may be caused by the highly polymorphic nature of potato. Alternatively, it may be caused by the fact that the monomorphic nature of these SSRs was based on a relatively small number of sequences (clusters containing less than 4 sequences from one or two genotypes), and/or a possible redundancy between clusters, as discussed above.

In some previous studies [3,7,8], it was observed that EST-SSRs had a lower level of polymorphism than genomic SSRs. Our study demonstrates that a large number of polymorphic SSRs can be retrieved from EST sequences, even in species that show low levels of polymorphism such as tomato (Table 1).

### Analysis of SSRs obtained with PolySSR

In general, ~5% of plant ESTs contain SSRs with a minimum repeat length of 20 nucleotides [3,10,30,31]. In mammals this proportion varies from 2% in sheep to 15.6% in mouse, while around 3.8% and 3.7% of chicken and zebra finch unique sequences contained SSRs [5]. In our results, 9% (*Brassica*) to 28% (rice) of the ESTs seem to contain SSRs. Also more than 13% of the non-redundant ESTs contained SSRs (Table 1), which is much higher than the published results. In rice non-redundant ESTs, 133,861 SSRs were detected. This is much more than the 48,351 SSRs found in the genomic sequence of rice subspecies *japonica *[26], but is less than the number predicted by SSRPrimer (425,432) [12]. Observed differences most likely result from the criteria used to identify SSRs, such as the minimum length of a SSR, whether or not only perfect repeats are considered, and whether or not mono-, penta and hexa nucleotide repeats are included [3]. In a previous study only di-, tri- and tetra- nucleotide perfect SSRs with at least 15 nucleotides were taken into account [26].

Some previous studies suggested that long SSRs are more frequently polymorphic [20,32], or that more alleles and larger PIC values should be expected [21,33,34] for long SSRs compared to short SSRs. Others observed no relationship between the repeat length and the informativeness of SSR [35-37]. Using PolySSR we have collected a large dataset with information on levels of polymorphism in several species that is ideally suited to address the controversy mentioned. Our data show that short SSRs are more frequently polymorphic than long SSRs in all species investigated. An explanation may be the classification of long and short SSRs. In this and other studies, long and short SSRs are selected on the basis of the repeat length in available sequences. These sequences may be from short or long alleles of a SSR, for example, a short SSR may be the shorter version of a long SSR. However, it can not be excluded that the relative low frequency of polymorphism observed in the long SSRs is the result of the poor clustering by CAP3. The fact that short SSRs are more frequently polymorphic demonstrates that the previous focus on longer SSRs expected to be more polymorphic, may have been incorrect. As a consequence many SSRs may have been excluded from further analysis and overlooked as potentially useful markers.

### SSR position in the expressed sequence

Most di- and pentanucleotide repeats are found in UTRs (Table 3), which is similar to earlier publications [10,18,20,27]. Most SSRs in coding regions are tri-nucleotide repeats, presumably because such SSRs do not cause frameshift mutations. We also studied the distribution of SSR polymorphisms across the ESTs and observed non-random patterns. In our study all species showed a higher SSR density in the 5' UTR than in the 3' UTR. This is similar to some published studies [27,38,39], but not others [3]. SSR polymorphisms in coding regions or in 5'UTRs may serve to modify expression or function of the genes with which they are associated. The number of repeats of SSRs in coding sequences may vary and thus providing a prolific source of quantitative and qualitative phenotypic variation [40]. Another interesting finding of our study is that a number of polymorphic SSRs is present on the translation start and stop sites. This was the case for all species studied. Such SSRs could have an impact on gene expression/translation and make it possible for the organism to adapt more easily to changing environmental conditions [40].

### Prospects

The only prerequisite for PolySSR is the availability of sufficient sequence information derived from different genotypes. It is especially in this area that that strong progress is likely to be made due to the introduction of new sequencing technologies [47], such as the 454 sequencing [48]. With these new tools fast amount sequencing of data can be generated from any given species in a rapid and cheap way, which will further increase the applicability of PolySSR. When 454 sequencing is carried out on cDNA derived from different genotypes, PolySSR can directly process the data, which are only a bit shorter than from traditionally sequenced ESTs.

Especially in agricultural crops were not so much activity is ongoing, new sequencing technologies in combination with PolySSR will revolutionarise SSR marker development. It is also anticipated that with the going down of prizes for sequencing, the technology will become affordable as well for ecological studies, where SSR markers are widely used for population analysis.

## Conclusion

The value of PolySSR is demonstrated by the fact that almost all tested SSRs predicted to be polymorphic were indeed validated as polymorphic. Large numbers of polymorphic SSRs were identified by PolySSR from publicly available EST sequences of potato, tomato, rice, *Arabidopsis*, *Brassica *and chicken. These results clearly demonstrate that ESTs are a valuable resource for developing polymorphic microsatellites. PolySSR supplies reliable polymorphic SSRs and high quality SSR primers, thereby decreasing the cost for designing and testing primers. In addition, PolySSR brings a novel approach employing the redundancy and heterozygosity of ESTs to develop markers based on SSRs that have been ignored in the past. In the PolySSR pipeline a primer design module is included that designs primers for amplification of the SSR taking into account the length of flanking sequences available and potential SNPs surrounding the SSRs. PolySSR has a broad applicability in non-model species when combined with the next generation of sequencing technologies.

The large number of SSRs detected with PolySSR allows a more in-depth investigation of general properties of SSRs. We show that short SSRs are more often polymorphic than long SSRs. In addition to identification of polymorphic SSRs and primer design, we used PolySSR to create a database of polymorphic SSRs. This database is available from the website [19].

## Methods

### Architectural structure

**Figure 2:**
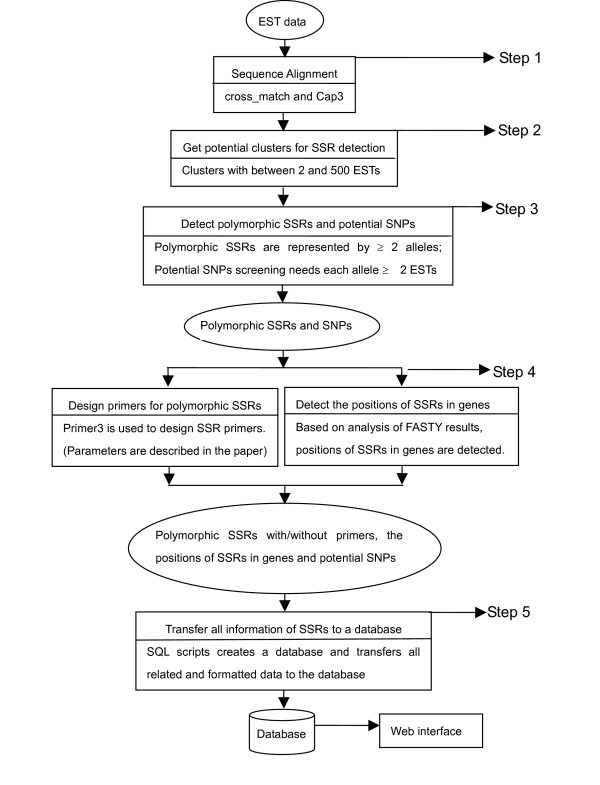
Flowchart of the PolySSR pipeline.

The PolySSR pipeline consists of five steps (Figure 2): 1) sequence alignment using cross_match [41] for removing vectors, and CAP3 [42] for sequence clustering; 2) selection of clusters with between 2 and 500 members; 3) detection of polymorphic SSRs and potential SNPs; 4) primer design for polymorphic SSRs using Primer3 [24] and detect SSRs positions on genes; 5) creation of a database for polymorphic SSRs and potential SNPs. This pipeline also includes a retrieval system (see the example website [19]). The pipeline is implemented in standard C-shell script while individual steps are written in the C language, with the exception of the sequence alignment tool (Perl), and the storage and retrieval systems (MySQL and PHP).

### Implementation

**Figure 3:**
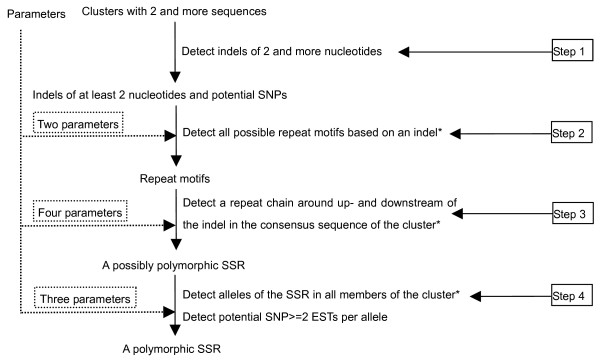
**Flowchart of the PolySSR core program**. Two parameters used in step 2 are the degree of matching in a repeat motif and the degree of matching in a repeat chain; four parameters used in step 3 include two parameters from step 2, and plus the length of flanking sequences of repeats and the minimum repeat times for different length of repeat motifs; three parameters used in step 4 consist of two parameters used in step 2 and the minimum number of sequences per allele. * actions in steps 2, 3 and 4 all use the algorithm described in Figure 4 and in the Materials and Methods section.

For detection of polymorphic SSRs, we use the method of matched filtering [43], which is based on the scheme that is described in Figure 3. The method of matched filtering is commonly used to process electronic signals; for example, it is used to catch perfect or imperfect matching signals and to filter out noise [43]. We use it to identify perfect and imperfect SSRs in DNA sequences. The process consists of four steps.

First, indels of at least two nucleotides are detected using all sequences present in a cluster obtained after CAP3 clustering (Figure 3). These indels may constitute polymorphisms of a putatively polymorphic SSR. Second, we detect all possible repeat motifs based on the sequence of the indel. Two thresholds for the degree of matching of a repeat motif and the degree of matching of a repeat chain (described later) are used to control the detection of perfect and imperfect repeats. Third, we detect the repeat of the motif in the DNA flanking the indel along the consensus sequence of the cluster constructed on the basis of the most frequent nucleotide per position by CAP3. Fifty nucleotides up- and downstream of the indel are considered. When the boundary of the repeat chain is outside of the 50 nucleotides-long string, the string is extended with an extra 50 nucleotides, if available. If a repeat chain with a minimum number of repeats is found neither up- nor downstream of the indel, the search is narrowed to a small region spanning the deletion, for example in sequences like CTGCTG***CTG. When up- and downstream sequences are combined, a SSR is identified that meets the demands for a minimum of 3 repeats. Besides the two parameters used in the second step two further parameters are used to identify a possibly polymorphic SSR: the minimum length of the flanking sequences of a repeat and the minimum repeat numbers for different motif lengths (2–6 nucleotides). Fourth, alleles are detected with different numbers of the sequential repeat motif. The two parameters used in the second step are used again in this step, and another parameter: the minimum number of sequences per allele is used to define reliable alleles. Polymorphic SSRs are positively identified when at least two reliable alleles are found in a cluster. Also in this step, potential SNPs are identified in the cluster when each allele of a SNP is found in at least two sequences.

**Figure 4:**
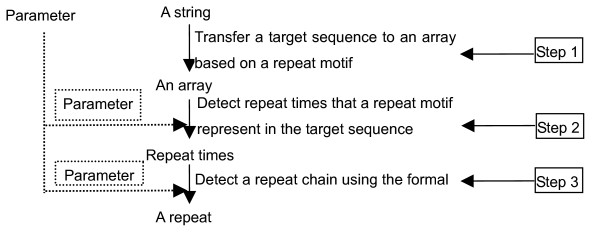
**The flowchart used to identify perfect and imperfect repeat chains**. The parameter used in step 2 is the degree of matching in a repeat motif; the parameter used in step 3 is the degree of matching in a repeat.

The second, third and fourth steps use the same algorithm to identify perfect and imperfect repeat chains from different sequences (indels, the consensus sequence and member sequences of a cluster). The algorithm to identify repeat chains consists of three steps (see Figure 4)

Step 1 obtains an array of match values from a motif and a target sequence.

The comparison starts from one end of the target sequence using the motif, and slides one nucleotide each step. The match value is the number of nucleotides matching the repeat motif. For example, for an indel CTG, CTG is one of the possible repeat motifs (CTG, CT and TG). If the upstream sequence of 50 nucleotides of the indel is CCTTTCTTCTACAACTACTACTGCTCCTCCTTATG**CTGCTGCTGCTGCTG**, then the array of match values will be 12110210200100200200**3**00201201210200**3**0 0**3**00**3**00**3**00**3**00.

Step 2 determines the total number of repeats of the motif in the target sequence, using a minimum threshold for the degree of matching in a motif.

*F*1 is a parameter specifying the minimum degree of matching in a repeat motif as a fraction between 0 to 1. Let *L *be the length of the motif; then L*F1 is the minimum match value for matching a repeat of the motif. *M *is the number of match values that is not smaller than the minimum match value in the target sequence. In the example, *L *is 3 for the repeat motif CTG; if we set *F*1 to 0.8, the minimum match value is 3*0.8 = 2.4; therefore the number of matching repeats (*M*) is 6, as there are six figures larger than 2.4 in array *Y*. Note that for motifs of length less than 5, an *F*1 of 0.8 requires exact identity; for a motif of length 5 or 6, one basepair mismatch would be allowed with this value of *F*1.

Step 3 determines the boundary of a repeat chain, based on the minimum threshold for the degree of matching in a repeat chain and the minimum threshold for the number of motif repeats in the chain.

In this step we take into account the number of nucleotides of the target sequence in which the first Z motif repeats are found, counting from the indel outwards. If the sequence contains non-matching segments interspersed between the Z motifs, the number of base pairs in which the first Z motif repeats are found (RL) is larger than Z·L, where L is the motif length. Parameter A is calculated for all Z as

$$A=\frac{Z\cdot L}{RL}$$

where Z ranges from the minimum number of motif repeats to the total number of motifs in the target sequence. A has a maximum value of 1.0 if no non-matching segments occur between the motif repeats, otherwise A < 1.0. *F*2 is an input parameter specifying the minimum threshold value for A, *i*.*e*. the minimum degree of matching in a repeat chain. The largest Z corresponding to an A ≥ *F*2 determines the length of the repeat chain. If no Z is found at least equal to the minimum number of repeats with an A ≥ *F*2, no repeat chain is detected.

If the end of the repeat chain is close (less than one repeat unit) to the end of the target segment, the segment is extended by 50 nucleotides based on the consensus sequence of the cluster, and the process is repeated.

### Parameters

In PolySSR, five criteria are used to identify perfect and imperfect polymorphic SSRs (Figure 3 and Figure 4): 1) the minimum degree of matching in a repeat motif (*F*1), 2) the minimum degree of matching in a repeat chain (*F*2), 3) the minimum length of the sequences flanking the SSR, 4) the minimum repeat times of di-, tri-, tetra-, penta- and hexanucleotide motifs, 5) the minimum of sequence redundancy per allele.

The degree of matching in a repeat motif *F*1 and the degree of matching in a repeat chain *F*2 are the important parameters to identify an imperfect SSRs. *F*1 allows mismatches to occur within a repeat motif. E.g. when *F*1 is 0.8, 20% of the nucleotides in a repeat motif may be mismatched (i.e. 1 in a penta- or hexanucleotide repeat, and 0 in shorter motifs). For instance, with *F*1 = 0.8, one mismatch is allowed in repeat motif ATGTA; in a target sequence ATGTTATGTAATGTA, an imperfect repeat ATGTT of ATGTA is identified in the string. The parameter *F*2 is used to identify imperfect SSRs with short non-matching sequences interspersed between the repeats. For example, the sequence ATGTAAATGTAATGTA contains three repeats of ATGTA and a one basepair (A) insertion. The *F*2 for the 3-repeat chain is calculated as 0.9 (*Z *= 3; *L *= 5; *RL *= 16; (Z * *L*)/*RL *= 15/16). If the threshold for *F*2 is set to 0.8, this imperfect chain is still accepted.

The length of the sequence flanking the SSR is an important factor for detecting reliable SSRs and getting sufficiently long sequences for designing primers. This is in particular important for ESTs, which are usually of poor quality especially at the beginning and end of the sequence. Also, if the repeat chain extends to the end of the sequence it might have been cut off at that point and the actual length is not known (Figure 1).

PolySSR allows users to set the minimum number of repeats to control the length of SSRs, with different minima for different lengths of the repeat motifs. If a repeat chain contains less than this minimum number of repeats, no SSR is recognized. Finally, the minimum sequence redundancy of each allele (the minimum number of sequences containing each allele) is used to define a reliable allele.

In this study these parameters were set as follows: the thresholds for *F*1 and *F*2 were 0.8, the minimum sequence length flanking the SSR was 25 nucleotides, and a minimum of 3 repeats for each repeat motif length; the minimum sequence redundancy per allele was set to 1 (i.e. no redundancy was required).

The criteria for primer design by Primer3 used in the pipeline were: the optimum size of the primers is 20 nucleotides with a maximum of 25 and a minimum of 18; the optimum temperature is 55°C, maximum of 65°C and minimum of 50°C and difference in temperature is 15°C; the maximum GC content is 80%, minimum GC 20% and the optimum 50%; the product size range is from 100 to 500 nucleotides.

### Characterization of polymorphic SSRs in genes

For the detection of polymorphic SSRs in coding regions, 5' or 3' untranslated regions (UTRs), or those containing a translational start (first codon) or stop (last codon) site (TSS) of a gene, two strategies can be used: alignment of a sequence with reference protein sequences, or ORF prediction using programs such as ESTscan (available in the TIGR Gene Index). We used the first method: FASTY was chosen as the tool to search the protein database rather than BLASTX, because it allows for frameshifts and produces better alignments with poor quality sequences [44]. The UniProt database was chosen as a reference database. The consensus sequences from the clusters with polymorphic SSRs were used to search the UniProt database (version of March 28th 2007) [45]. An in-house parsing program contained as an additional program in the PolySSR pipeline, is used to analyze the FASTY results, together with the alignment information, the potential indels, and SSRs information. The parsing program first sorts the FASTY results by *E*-value to get the sequence with the highest similarity to the consensus sequence. Next, any frameshift in the sequence is detected and corrected based on the potential indels, after which the best translation is detected. Finally, SSRs in coding, 5'or 3' UTRs and TSS are identified and the lengths of coding, 5' or 3' UTRs are accumulated. Based on that, the densities of SSRs in coding, 5' and 3'UTRs are calculated.

### Other programs

The program Sputnik [13] was used to identify all SSRs present in the EST sequences.

### ESTs used

All potato ESTs with cultivar information, in total 219,765 reads obtained from the EMBL database [46] (version 88) were used to detect polymorphic SSRs. EST sequences of other species were also used to detect polymorphic SSRs.: 734,275 *Arabidopsis *ESTs, 1,211,078 rice EST, 599,330 chicken ESTs, 163,750 *Brassica *ESTs and 249,794 tomato ESTs with genotype information were obtained from the EMBL database [46] (version 89).

### Checking the uniqueness of a SSR

A program called CheckSSR was developed to check whether SSRs in our SSR database are really unique SSRs. It does this by taking the primers of these SSRs and searching for matches in our sequence database (the consensus sequences of clusters and singletons). When either the forward or reverse primer is found in other sequences, it should be excluded from marker development, as problems might be expected during PCR amplification.

### Validation of SSRs

Putative polymorphic SSRs of potato were validated on a small set of cultivars including Kennebec, Shepody and Bintje (the cultivars from which most of the ESTs in the database were derived), supplemented with the cultivars Katahdin and Kuras.

Each SSR was amplified in a 10 μl PCR reaction that included ~20 ng DNA, 1× PCR buffer, 2.5 mM MgCl_2_, 0.2 mM dNTPs, 100 nM reverse primer, 50 nM of unlabelled forward primer, 50 nM fluorescent labelled forward primer (FAM-6, HEX or NED) and 0.3U DNA polymerase. The reaction conditions were: 94°C for 2 min followed by 30 cycles of [94°C for 1 minute, 58°C for 2 minutes, 72°C for 1 minute 30 seconds] and a final extension step of 72°C for 5 minutes. Initially, 8 μl of the PCR reaction was size separated using agarose gel electrophoresis. The size of the PCR products was estimated using the 100 bp or 500 bp molecular ladders (Invitrogen) as a reference. Those assays that produced amplification products of less than 500 bp were re-amplified and 1 μl of the PCR reaction was purified using an ethanol precipitation. The products were size separated along with the Genescan-400HD size ladder (Applied Biosystems), by capillary gel electrophoresis using the ABI3100 Genetic Analyser (Applied Biosystems). The peak heights and fragment sizes were analysed using GeneMarker (SoftGenetics).

To study the relationship between the rate of polymorphism and the length of SSRs, the polymorphic potato SSRs selected for validation were divided into two groups: the long and short SSRs. The long SSRs are SSRs on the consensus sequences with at least 10 repeats of a repeat motif for a di-nucleotide SSR, 6 repeats for tri-nucleotide SSR, 5 repeats for a tetra-, penta- or hexanucleotide SSR. The short SSRs are SSRs with a maximum of 5 repeats for a di-nucleotide SSR, and 4 repeats for tri-, tetra-, penta- and hexa-nucleotide SSR. The minimum number of repeats was in this case set to 3 for all SSR types.

For tomato, we used SSRs from the literature [21] for validation, as this study also includes information on monomorphic SSRs present in tomato. Of the 75 EST-derived SSRs used in the study, 32 were validated by the authors as polymorphic and 43 as monomorphic. CheckSSR was used to identify these in our database.

## Authors' contributions

BV, HE and JT identified the need to develop the program; JT designed the algorithm and wrote the source code. SB and JJ did the experimental validation. All authors participated in the drafting of the manuscript and approved the final version.

## Supplementary Material

Additional file 1 Supplementary table. Click here for file
