# Supplementary material for: Large-scale identification of polymorphic microsatellites using an in silico approach
Source: BMC Bioinformatics. 2008 Sep 15;9:374. doi: 10.1186/1471-2105-9-374 (PMC2562394; doi:10.1186/1471-2105-9-374)
Supplement: Additional file 1 — Supplementary table. [file 1471-2105-9-374-S1.doc]

Additional file 1

| Primer name |  | Sequence name in TIGR database | Repeat Motif | Primer Sequence(5'-3') | Annealing Temperature | Polymorphic (Y,N) or remark | Observed number of alleles |
| --- | --- | --- | --- | --- | --- | --- | --- |
| st572f^1^ | LP | potato|TC112831 | CAG | ATCTAACCGAGGAGGGTTGC | 58 | Y (Bintje and Katahdin present 7 alleles, others present 8 alleles) |  |
| st572r |  |  |  | TCAACAGCTGGAGCATCATC | 58 |  |  |
| st2663f | LP | potato|TC115448 | TA | CCTCCTCCTCATCATGCACT | 58 | Y | 4 |
| st2663r |  |  |  | ACACCCTCCTCTCCCAAAAA | 58 |  |  |
| st4519f | LP | potato|TC129885 | AGG | CGTGAGGCAAATGCTACTGA | 58 | around 850 base pairs |  |
| st4519r |  |  |  | CCTTGCCCTCTTTGCTGTAG | 58 |  |  |
| st4848f | LP | potato|TC112792 | TA | GGTGCTTGGGTTCTCAATTC | 58 | Y | 7 |
| st4848r |  |  |  | CACGTCAAGACAAAGACACAA | 58 |  |  |
| st8124f | LP | potato|TC113539 | TCT | TCACACCAAAACCCATTTCA | 58 | Y | 3 |
| st8124r |  |  |  | CTCAGGGACTCCAAGTCCAA | 58 |  |  |
| st8313f | LP | potato|TC127116 | CTT | TTGATATTAACCATGGCAGCA | 58 | Y | 2 |
| st8313r |  |  |  | TTGATGGGATTGCACAGAAA | 58 |  |  |
| st8366f | LP | potato|TC113993 | CAA | CGCAACAGCAACAACAACTT | 58 | Y | 4 |
| st8366r |  |  |  | GGCAGGAGTCTTATCGTTGG | 58 |  |  |
| st8474f | LP | potato|TC129379 | AAG | GCTGCTAAACACTCAAGCAGAA | 58 | Y | 7 |
| st8474r |  |  |  | GCTGAAAACAGCTAAATCCCA | 58 |  |  |
| st11672f | LP | potato|TC127996 | CAA | CCTTTTGCTTTGAACCCATC | 58 | Y | 3 |
| st11672r |  |  |  | CTGCTTGAAGTTCTGCTGGA | 58 |  |  |
| st12061f | LP | potato|TC126746 | AT | TCGTCCTTCTAATTCATTATCCAA | 58 | Y | 4 |
| st12061r |  |  |  | TGGGGATAAATTGTTGTGGG | 58 |  |  |
| st12951f | LP | potato|TC129417 | CAG | GTCGAAAGCAGAAGGCTACG | 58 | Y | 6 |
| st12951r |  |  |  | TTGCGACTGCTGTGGATTAC | 58 |  |  |
| st13188f | LP | potato|TC119291 | TA | GCTACGCTCCTTGAGTGGTC | 58 | Y | 4 |
| st13188r |  |  |  | GCCAACCATTTCAATACAAGC | 58 |  |  |
| st13237f | LP | potato|TC125891 | ATA | AAGAAACTGAGTTGTGTTTGGGA | 58 | Y | 3 |
| st13237r |  |  |  | CAGGTGGCTTGATTGGATCT | 58 |  |  |
| st14481f | LP | potato|TC119491 | TTTG | CCAAGGAAGGTCATTGGAGA | 58 | Y | 4 |
| st14481r |  |  |  | TGTTCCCTAAATGAGGCTGG | 58 |  |  |
| st15682f | LP | potato|TC113652 | AT | TCGAGATGCAGTGTGGAGTC | 58 | Y | 6 |
| st15682r |  |  |  | TCATTCGGCTTGAATCATTG | 58 |  |  |
| st15928f | LP | potato|TC120214 | GGT | CGCCATTTTCCTTCTTCTTC | 58 | Y | 2 |
| st15928r |  |  |  | CTCAGGCTCCAATCCTTCAC | 58 |  |  |
| st17019f^3^ | LP | potato|TC112734 | AAT | GACGCAGAACTCATCTTGTTCA | 58 | Y | 3 |
| st17019r |  |  |  | GCGATTCGATTGCATTCTCT | 58 |  |  |
| st17765f | LP | potato|TC112793 | TA | CAAGCACGTTACAACAAGCAA | 58 | Y | 4 |
| st17765r |  |  |  | CATGATAAGCAGCTCCAACG | 58 |  |  |
| st18034f | LP | potato|TC119770 | TTC | TTTGTGCTCAACTTTCCATGTC | 58 | Y | 3 |
| st18034r |  |  |  | GCCAAAAAGAGAAACCCCAT | 58 |  |  |
| st18036f | LP | potato|TC114120 | TCT | AAACATGGCGCAAATGAAG | 58 | Y | 4 |
| st18036r |  |  |  | CGCCAAATTAGGGAATGAGA | 58 |  |  |
| st19719f | LP | potato|TC127716 | TTG | CAATATGAAGGCCGGAGAGA | 58 | Y | 5 |
| st19719r |  |  |  | ACCCATATTTCCAAGGCACA | 58 |  |  |
| st22164f | LP | potato|TC123357 | TTC | TCTCAATGGCTTCTCCTTCC | 58 | No products |  |
| st22164r |  |  |  | TAGAATTGCCCAAATCCAGG | 58 |  |  |
| st22890f | LP | potato|TC114376 | GTT | GAGATGAAGGTTTTTGCGTTG | 58 | No products |  |
| st22890r |  |  |  | CCCATCTTTTGAAAAACCCA | 58 |  |  |
| st24630f^3^ | LP | potato|TC119168 | ATCT | CCCCCAGTGGTTTCACATAC | 58 | Y | 4 |
| st24630r |  |  |  | TGGGAAAAGGTACAAAGACGA | 58 |  |  |
| st25030f | LP | potato|TC114108 | CAC | ACAGCAACATTTCCAAAGGG | 58 | Product about 4.5k base pairs |  |
| st25030r |  |  |  | TCCTGAGGATGATTTCCCTG | 58 |  |  |
| st1447f^3^ | SP | potato|TC119255 | GAA | CGGGGGACTCCATAACCTAT | 58 | Y | 2 |
| st1447r |  |  |  | GACTGATTTAGGCTGCTGGC | 58 |  |  |
| st2696f | SP | potato|TC112046 | TCT | GAAACCTACTCGTCCCGTTG | 58 | Y | 2 |
| st2696r |  |  |  | TCTCACAATGGGGTTGATGA | 58 |  |  |
| st3941f | SP | potato|TC119506 | TGA | ATTGGCAAGAAAGGAAGCAA | 58 | Y | 5 |
| st3941r |  |  |  | TCTGGGGTTTTCAAGCATTC | 58 |  |  |
| st4149f | SP | potato|TC126427 | AT | AAAATTATGTGCCGAGTCCG | 58 | Y | 3 |
| st4149r |  |  |  | GGAGGGGAGAAATCAAATCA | 58 |  |  |
| st4562f | SP | potato|TC113691 | GAA | AAAAGAGGAGGATGGGGAGA | 58 | Product about 800 base pairs |  |
| st4562r |  |  |  | AACCGAGCATTCATTCCTTG | 58 |  |  |
| st4913f^2^ | SP | potato|TC119143 | TGG | CAGGGAGAAGTTGGCTTCAG | 58 | Y | 5 |
| st4913r |  |  |  | GGAACAGGGCCTTTCATACA | 58 | link to cluster 3805 |  |
| st5747f | SP | potato|TC119692 | GCA | GGAAAATCACCATCACCACC | 58 | Product larger than 500 base pairs |  |
| st5747r |  |  |  | TGTACCGACAACTCGCTCTG | 58 |  |  |
| st5843f^3^ | SP | potato|TC127857 | AGA | CGGATAATGAAGCTGGTTCTG | 58 | Y | 4 |
| st5843r |  |  |  | TGGCCTTCTTCAAGACTGGT | 58 |  |  |
| st6851f | SP | potato|TC126733 | CTT | GGCAAAGAGGCTGAAAAGTG | 58 | no clear products |  |
| st6851r |  |  |  | GGTAAGCTGATTGCTCTGGC | 58 |  |  |
| st7676f | SP | potato|TC120021 | CAG | TGCTTTTTCCTGGGGATATG | 58 | no products |  |
| st7676r |  |  |  | TGTGATTGTTGCAACGGAAT | 58 | link to cluster 24551 |  |
| st7960f | SP | potato|TC126206 | CT | GGTGATGATCTCAATGCGAG | 58 | Y | 2 |
| st7960r |  |  |  | TGGGACCCAAAAACATTCAT | 58 |  |  |
| st7982f | SP | potato|TC127369 | TCT | CAAACACCCAAATGGCTTCT | 58 | Y | 3 |
| st7982r |  |  |  | AGCATAGATGGTATTGCCCA | 58 |  |  |
| st10536f | SP | potato|TC127046 | CGG | AAACCCTTGTCGTCACTGCT | 58 | no products |  |
| st10536r |  |  |  | GAAAGAATTGTGCCGTCGAT | 58 |  |  |
| st11129f | SP | potato|TC126571 | ACC | ACTACCACCACCTCCACCAC | 58 | Y | 2 |
| st11129r |  |  |  | TCCGGCGTGCTATACTTTCT | 58 |  |  |
| st11361f | SP | potato|TC119446 | CAC | AACACCACCGCCACTAACTC | 58 | Y | 2 |
| st11361r |  |  |  | ATATGACGATGACGTGGCAA | 58 |  |  |
| st11378f | SP | potato|TC119875 | TTC | TCTTCAGGCAATTCCATGTG | 58 | Y | 4 |
| st11378r |  |  |  | CACTTCCCTTCTCCATTTCG | 58 |  |  |
| st11889f | SP | potato|TC112779 | GCC | GTACACCCAAAACCGGCTAA | 58 | Y | 4 |
| st11889r |  |  |  | CTTTGTCTGGATCGTGCTCA | 58 |  |  |
| st11944f^3^ | SP | potato|TC113871 | CCG | TTCTTCAATGGCAGAAAGGG | 58 | Y | 3 |
| st11944r |  |  |  | GAGTTTGGCGGAACGAGTAG | 58 |  |  |
| st13093f^2^ | SP | potato|TC119016 | TCT | GTCTTGATAAACGCCGTCCT | 58 | N |  |
| st13093r |  |  |  | GCTCTAGGCACATGCTTTCC | 58 | link to st15317 |  |
| st14911f | SP | potato|TC126648 | TCT | GGAGGTTTCAGTGATGGGAA | 58 | Y | 4 |
| st14911r |  |  |  | TCAAAGACTTGGAACGCAAT | 58 | link to st18454 |  |
| st16611f | SP | potato|TC112235 | TAT | TTGGGTTATGAGTTCCTGCC | 58 | no clear products |  |
| st16611r |  |  |  | AACACAGGTTGAAGATCGGG | 58 |  |  |
| st19532f | SP | potato|TC128796 | AT | CAATGAATGCTGGTGCACTT | 58 | Y | 4 |
| st19532r |  |  |  | CAATGAGAGGGGGAGTCAAG | 58 |  |  |
| st21186f^1^ | SP | potato|TC112849 | GGT | GGATGGCCCTACTTCATTCA | 58 | Y (Bintje, Katahdin present 6 alleles) | 6 |
| st21186r |  |  |  | TCTTTCCTTGGGACTGATGG | 58 |  |  |
| st21593f^1^ | SP | potato|TC114725 | TA | TGGACCTACAGGTTCCGTTC | 58 | Y (Bintje,Katahdin,Kennebec,Shepody present 8-14 alleles) |  |
| st21593r |  |  |  | CCCTTAACTCGTAAAATATCCCAA | 58 |  |  |
| st24562f | SP | potato|TC127690 | TC | GAAAGGGATCGTGTCCATGT | 58 | Y | 3 |
| st24562r |  |  |  | TTCATACAACAGTGAATGTGCAA | 58 |  |  |
| st778f | LN | potato|TC118480 | ATA | CCACGGATGGAACCAATTTA | 58 | Y |  |
| st778r |  |  |  | CTTCACCTCCTGCCTTTCTG | 58 |  |  |
| st3772f | LN | potato|TC132254 | AAT | AGGATCGGTGCGTGAAATAC | 58 | Y |  |
| st3772r |  |  |  | GCAGAAAGCAAAGCAGGAGA | 58 |  |  |
| st3797f | LN | potato|TC122873 | AT | CAACGCTACTCAATGGCTCA | 58 | no clear products |  |
| st3797r |  |  |  | ACAACTCTAGAACGAGAGGAACA | 58 |  |  |
| st4412f | LN | potato|TC123932 | CAACT | TTCAGCTCAGTTTGACTCGC | 58 | no products |  |
| st4412r |  |  |  | TCCTCGGGCATACGAATATC | 58 |  |  |
| st8580f | LN | potato|TC114412 | CTCC | ACTGCCGCAAAAAGTGAAAA | 58 | Y |  |
| st8580r |  |  |  | GCCGCTAGGTGGAGTAGATG | 58 |  |  |
| st10066f^1^ | LN | potato|TC113209 | AAG | CATTGGCAACATCACCATTC | 58 | Y (both Kennebec and Shepody show 5 alleles) |  |
| st10066r |  |  |  | TGTCGATTTTGTATCCGTGAA | 58 |  |  |
| st10460f | LN | potato|TC112472 | TCT | GCCAGACTTGCCATTTCATT | 58 | no products |  |
| st10460r |  |  |  | CCAAGCGAACCAAAATAGGA | 58 |  |  |
| st11061f | LN | potato|TC121499 | CGC | CCCAGTTCGCCTCTCTAATG | 58 | Y |  |
| st11061r |  |  |  | GGGAGTTGAGATGCGAGAAA | 58 |  |  |
| st11363f | LN | potato|TC114005 | GGA | AGTTATCGTGAATGGAGCCG | 58 | Y |  |
| st11363r |  |  |  | GAGGATCCCAAACCACAAAA | 58 |  |  |
| st12560f | LN | potato|TC126302 | CGG | GGACATGAGGAGAGGCAGAG | 58 | one product larger than 500 base pairs |  |
| st12560r |  |  |  | CGCTTCCTAACTCCACGAAA | 58 |  |  |
| st14058f | LN | potato|TC128114 | TGA | TTTACTACCCTCATGCAACGC | 58 | no products |  |
| st14058r |  |  |  | CGCCGGTAAAGCTTCAATAA | 58 |  |  |
| st15123f | LN | potato|TC112534 | AC | TTTGTTCCCACCTCACAACA | 58 | Y |  |
| st15123r |  |  |  | TGGGGTAACAGCAAAAGAGC | 58 |  |  |
| st15797f | LN | potato|TC113541 | TAT | AAGGTGTTTGCCCATGCTAC | 58 | no clear products |  |
| st15797r |  |  |  | CAGCTCTCATATCGGGGAAA | 58 |  |  |
| st18083f | LN | potato|CV302432 | AGC | TCCTGTTGAGAAAGAGCCGT | 58 | N |  |
| st18083r |  |  |  | TTAAAAGTTGTTGGGCGAGG | 58 |  |  |
| st21789f | LN | potato|TC122491 | ATA | GGAAATGGTTGAGGCAAATG | 58 | no products |  |
| st21789r |  |  |  | CAGCAAATGGCTTTACAAACA | 58 |  |  |
| st1962f | SN | potato|TC132429 | CAA | TCCACAACTACCCTCCAATTC | 58 | Y |  |
| st1962r |  |  |  | TCCAGATGCTTGAGTTGCAC | 58 |  |  |
| st2241f | SN | potato|TC112031 | AAC | CGGTTTTGGAGGAGAAATCA | 58 | N |  |
| st2241r |  |  |  | CGTCATCTGCAGAGGCATAA | 58 |  |  |
| st2988f^2^ | SN | potato|TC117181 | ATG | GGGAATGGAAATGCAACACT | 58 | Y |  |
| st2988r |  |  |  | CGAGGGTAGTGAGGATGAGC | 58 | link to cluster 22236 |  |
| st7428f | SN | potato|TC128821 | CT | TCATTCTCTCCACGTTTCCC | 58 | Y |  |
| st7428r |  |  |  | GCTTCGGAAGTGGATCAGAG | 58 |  |  |
| st8084f | SN | potato|TC114075 | ATG | TTCCACCTTCATCTTTCTCCA | 58 | Y |  |
| st8084r |  |  |  | GACCATCACCCCAAGCTAAA | 58 |  |  |
| st11431f | SN | potato|TC112606 | GCA | GCAACAAGGAGTGAGTGCTG | 58 | larger products 900 base pairs |  |
| st11431r |  |  |  | GGTGGATAAGGTGGTGATGG | 58 |  |  |
| st11600f | SN | potato|TC119239 | CAA | AACCCCATCAACCCTACTCC | 58 | Y |  |
| st11600r |  |  |  | GAAGATGAGGAAGCACCAGC | 58 |  |  |
| st11798f | SN | potato|TC112474 | TCT | GCCAGACTTGCCATTTCATT | 58 | Y |  |
| st11798r |  |  |  | TAAGTTCCAGCATCATGCCA | 58 |  |  |
| st12036f | SN | potato|TC120131 | AAC | TCATCGGTATACCTCTCGCC | 58 | Y |  |
| st12036r |  |  |  | GTCGTTAGCCGGGTTGTAAA | 58 |  |  |
| st15317f^2^ | SN | potato|TC119016 | TCT | GTCTTGATAAACGCCGTCCT | 58 | N |  |
| st15317r |  |  |  | GCTCTAGGCACATGCTTTCC | 58 | link to st13093 |  |
| st15396f | SN | potato|TC112118 | CT | TTTTCCCTGGAAATCGAAGA | 58 | no products |  |
| st15396r |  |  |  | GCTCAGCTTCAGTTGGGTTC | 58 |  |  |
| st18454f^2^ | SN | potato|TC126648 | TCT | GGAGGTTTCAGTGATGGGAA | 58 | Y |  |
| st18454r |  |  |  | TCAAAGACTTGGAACGCAAT | 58 | link to st14911 |  |
| st19682f | SN | potato|TC127657 | AAG | CATACCAAGGGAATGATGGG | 58 | Y |  |
| st19682r |  |  |  | ATGAGAACTGCCTGCCAACT | 58 |  |  |
| st21669f | SN | potato|TC132161 | ATC | CTCATCGTGCTTTCACCTCA | 58 | Y |  |
| st21669r |  |  |  | GGAGACTTACGTCGGTCGAG | 58 |  |  |
| st24923f | SN | potato|TC128807 | ACA | CTCAAACTCCTGCCAAGGAA | 58 | clear products about 500, 600 base pairs |  |
| st24923r |  |  |  | TCGGAGAAATCATTGTTGTTG | 58 |  |  |

LP = long polymorphic SSR; SP = short polymorphic SSR; LN = long non-polymorphic SSR; SN = short non-polymorphic SSR.

Primers that produced no product, no clear product or a product larger than 500 base pairs were not tested further. SSR markers that were polymorphic (Y) or monomorphic (N) from the experimental results are indicated. For the validation experiment 5 cultivars (Kennebec, Shepody, Bintje, Katahdin and Kuras) were used.

SSR signed by ^1^ have produced more than 4 alleles in some genotypes

SSR signed by ^2^ have been discovered in another cluster as well.

SSR signed by ^3^ are not in our database anymore.

Average number of alleles for long SSRs is 4.1 and that of short SSRs is 3.4, excluding SSRs most likely targeting paralogs,e.g. showing more than 4 alleles in one variety
